# Evaluation of prognostic scoring systems in liver cirrhosis patients with bloodstream infection

**DOI:** 10.1097/MD.0000000000008844

**Published:** 2017-12-15

**Authors:** Hong Zhao, Xiuling Gu, Ruihong Zhao, Yu Shi, Jifang Sheng

**Affiliations:** a State Key Laboratory for Diagnosis and Treatment of Infectious Diseases, Collaborative Innovation Center for Diagnosis and Treatment of Infectious Disease, The First Affiliated Hospital, Zhejiang University School of Medicine, Hangzhou; b Department of infectious diseases, Cixi People's Hospital, Cixi, China.

**Keywords:** bloodstream infection, cirrhosis, prognostic models

## Abstract

Patients with cirrhosis are at increased risk of developing bloodstream infections (BSIs), and the short-term mortality rate in those patients is high. The aim of this study was to compare the different scoring models to predict mortality in cirrhotic patients with BSIs.

A total of 222 cirrhotic patients with BSIs were retrospectively included in the study. The demographic, clinical, and microbiologic data were collected and patients were followed for at least 28 days after blood cultures were established. A multivariable Cox proportional hazard model was used to identify independent risk factors for 28-day all-cause mortality. The prognostic accuracy of different scoring models (chronic liver failure-organ failure [CLIF-OF], model for end-stage liver disease [MELD], systemic inflammatory response syndrome [SIRS], and Pitt scores) were compared with the C-index and receiver operating characteristic curve (ROC).

Forty deaths were recorded on day 28 after blood cultures were established. Male sex (hazard ratio [HR] = 2.75, 95% confidence interval [CI] = 1.10–6.86), international normalized ratio (INR) (HR = 1.76, 95% CI = 1.35–2.30), serum bilirubin (HR = 1.002, 95% CI = 1.000–1.003), circulation failure (HR = 3.56, 95% CI = 1.63–7.79), lung failure (HR = 2.23, 95% CI = 1.09–4.57), and non-primary BSI source (HR = 2.27, 95% CI = 1.09–4.73) were identified as independent risk factors for mortality in cirrhotic patients with BSIs. In predicting 28-day mortality, CLIF-OF and MELD scores had significantly high C-indices (0.79 and 0.76, respectively) and ROC values (0.786 and 0.782, respectively) compared with Pitt and SIRS scores (C-indices: 0.61 and 0.57, respectively; ROC values: 0.591 and 0.637, respectively).

Cirrhotic patients with BSIs had high short-term mortality rates. Our data suggested that both CLIF-OF and MELD scores can be used to predict the short-term prognosis of these patients.

## 1 Introduction

Patients with cirrhosis have an increased risk of developing infections.^[1]^ Bloodstream infections (BSIs) are a common type of infection in patients with cirrhosis.^[2]^ It has been reported that BSIs occur in 4% to 21% of cirrhotic patients, with a 10-fold higher incidence than in non-cirrhotic patients.^[3]^ The severity of BSIs is also higher in patients with cirrhosis who are more likely to die from sepsis than individuals without cirrhosis.^[1,4]^ In several studies, including general patients with bacteremia, cirrhosis was shown to be an independent predictor of mortality.^[5,6]^ Early recognition of the severity of infections in patients with cirrhosis is important for decision-making in clinical practice. Currently, 4 scoring systems are available for predicting mortality in cirrhotic patients with BSIs, as follows: scores for BSI severity, such as the Pitt score^[7,8]^; systemic inflammatory response syndrome (SIRS) criteria for defining severe sepsis^[9]^; cirrhosis-specific scores, such as Model for End-Stage Liver Disease (MELD)^[10]^; and organ failure scores, such as chronic liver failure-organ failure (CLIF-OF).^[11]^ Therefore, the aim of this study was to identify the optimal scoring system for prediction of short-term mortality among cirrhotic patients with BSIs.

## 2 Patients and methods

### 2.1 Patients

We retrospectively enrolled cirrhotic patients who developed BSIs from December 2010 to December 2016 in Department of Infectious Diseases of the First Affiliated Hospital of Zhejiang University. For those patients experiencing multiple episodes of BSIs during the study period, only the first BSI was included for analysis. The study fulfilled the principles of the Declaration of Helsinki and was approved by the Ethics Committee of The First Affiliated Hospital of Zhejiang University. Written consent was obtained from each participant or their legal representatives. Patients with disseminated malignancies were excluded.

### 2.2 Definitions

Cirrhosis was diagnosed by liver biopsy, endoscopic signs of portal hypertension, radiologic evidence of liver nodularity, or clinical evidence of prior hepatic decompensation, including ascites, hepatic encephalopathy, and upper gastrointestinal bleeding, in patients with chronic liver diseases^[12]^ ascites was diagnosed by clinical examination and confirmed by ultrasonography. Hepatic encephalopathy was defined and graded by the West-Haven criteria. Organ failure was defined by the CLIF-OF score, as follows: liver failure, serum bilirubin ≥12 mg/dL or 204 μmol/L; coagulation failure, international normalized ratio (INR) ≥2.5; kidney failure, creatinine ≥2.0 mg/dL or 176 μmol/L; cerebral failure, hepatic encephalopathy grade III or IV by the West-Haven criteria; circulation failure, need for vasoactive agents; and lung, PaO_2_/FiO_2_ ≤200 or SpO_2_/FiO_2_ ≤214. Acute-on-chronic liver failure (ACLF) was diagnosed when patients had ≥2 organ failures.^[13]^ BSI was defined as the growth of a non-common skin contaminant from P1 BCs, and of a common skin contaminant from at P2 BCs drawn on separate sites with signs of infection.^[14]^ The primary source of BSI was defined according to the Centers for Diseases Control and Prevention criteria.^[15]^ When the same microorganism was isolated from the blood cultures and other sites, the BSI was considered to be secondary; otherwise, the BSI was considered to be primary. Polymicrobial bacteremia was defined as ≥2 microorganisms recovered from the blood cultures. Other sites of infections were defined as follows: pneumonia, new pulmonary infiltrate with fever, respiratory symptoms, findings on auscultation, or WBC count >12,000/mm^3^ or <4000/mm^3^; spontaneous bacterial peritonitis (SBP), polymorphonuclear cells in ascitic fluid >250/μL; urinary tract infection, urine WBC >10/high power field with positive culture and symptom of urinary irritation; and other bacterial infections included skin and intra-abdominal infections. A community-acquired (CA) infection was defined as an infection contracted outside of the healthcare setting or an infection present within 48 hours after hospitalization.^[16]^ A healthcare-associated (HCA) infection was defined as an infection within 48 hours of hospital admission in patients within a healthcare environment (e.g., hospitalization or hemodialysis clinic, intravenous chemotherapy during the past 1 month, admission for at least 2 days, or underwent surgery in the past 6 months, or residence in a nursing home or a long-term care facility; 18). A hospital-acquired (HA) infection was defined as an infection diagnosed 2 days after admission to a healthcare facility or hospital.^[17]^

### 2.3 Treatment

#### 2.3.1 Empiric antimicrobial therapy

Generally, the initial empiric antimicrobial therapy was designed against Gram-negative BSIs, which included fluoroquinolones, β-lactam/β-lactamase inhibitors, and third- or fourth-generation cephalosporins and carbapenems. If patients were at risk for resistant Gram-positive bacteria (e.g., *Enterococcus faecium* or methicillin-resistant *Staphylococcus aureus* [MRSA]), vancomycin, or teicoplanin was added. Anti-fungal drugs were usually not included as part of empiric antimicrobial therapy. In the current study, all patients received initial empiric antimicrobial therapy within 24 hours after blood for cultures were drawn. Therefore, the initial empiric antimicrobial therapy was considered adequate if the therapy included at least one antibiotic that was active in vitro against the causative microorganisms and the dosage and route of administration conformed with current medical standards.

#### 2.3.2 Treatment of complications of cirrhosis

Hepatitis B virus (HBV)-associated liver cirrhosis (LC) patients with detectable HBV-DNA received nucleoside analog treatment. Overt ascites was treated with diuretics and intravenous albumin and large or refractory ascites was treated with paracentesis plus intravenous albumin. Renal failure was treated with intravenous albumin with terlipressine. Hepatic encephalopathy was treated with lactulose, antibiotics, and L-ornithine aspartate. Circulation failure was treated with fluid replacement, followed by vasoactive agents. Respiratory failure was treated with oxygen therapy or mechanical ventilation.

#### 2.3.3 Calculation of scoring systems

The Pitt score (16) was calculated as follows: oral temperature (2 points for ≤35 °C or ≥40 °C, 1 point for 35.1–36.0 °C or 39.0–39.9 °C, and 0 points for 36.1–38.9 °C); acute hypotension (2 points for a decrease in systemic blood pressure >30 mmHg and a decrease in diastolic blood pressure >20 mmHg); ventilator use (2 points); heart failure (4 points); and consciousness (0 points for alertness, 1 point for disorientation, 2 points for stupor, and 4 points for coma). The SIRS score (13) was calculated as follows: 1 point for a core temperature >38 °C or <36 °C; heart rate >90 bpm; respiratory rate >20 bpm; and white blood cell (WBC) count >12,000/mm. The MELD score (6) was calculated using the following formula: 9.6 × log_e_ (creatinine [mg/dL]) + 3.8 × log_e_ (bilirubin [mg/dL]) + 11.2 × log_e_ (INR) + 6.43 × (etiology: 0 if cholestatic or alcoholic, otherwise 1). The CLIF-OF score (5), with a range of 0 to 18, is proposed to evaluate organ failure in ACLF patients.

### 2.4 Data collection

We collected the following clinical and demographic information in a prespecified datasheet. We collected the following data: age; sex; presence of diabetes; etiology of cirrhosis; prior decompensation history and events; decompensation events for the current hospitalization; and severity of cirrhosis at the time of admission. Prognostic scores were recorded on the day of BSI onset. Epidemiologic classification of BSI, BSI source, and microbiology records were also documented. The 28-day mortality rate was verified by medical records, telephone contact, or an in-person visit. Survival time was calculated from the time blood was drawn for culture.

### 2.5 Statistical analysis

Continuous variables are expressed as the mean ± standard deviation (SD) or median with interquartile range, and were compared with a Student *t* test or Mann–Whitney *U* test. Nominal variables are expressed as the number (percentage) and compared using a chi-squared test. The performance of several prognostic models was measured by the C-index (R version 3.1.2; The R Foundation for Statistical Computing) and the C-index was compared using a *t* test. The receiver operating characteristic curve (ROC) was calculated and compared by a Z test (MedCalc Software, Belgium). In addition, the sensitivity, specificity, positive predictive value, and negative predictive value at an optimal cut-off point of the model score was compared among different scoring models.

## 3 Results

### 3.1 Clinical characteristics, etiology, and mortality

**Figure 1:**
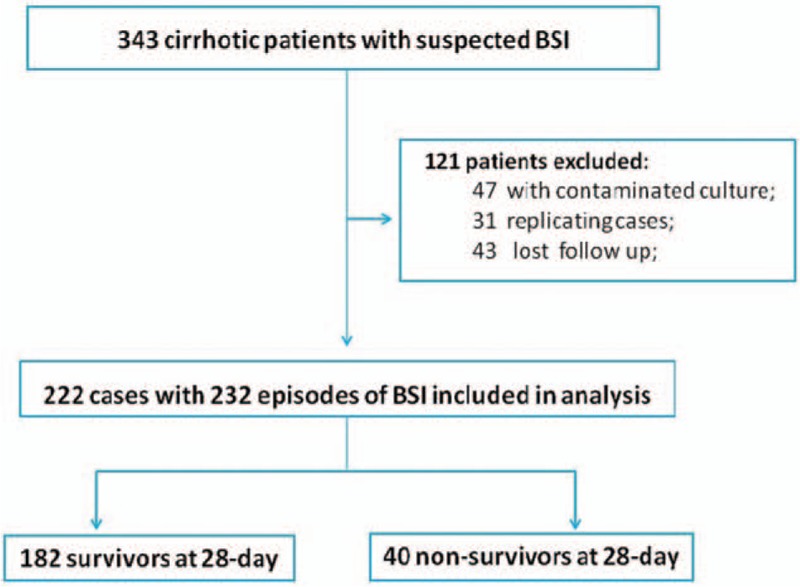
Flow chart of patient selection in the study.

Three hundred twenty-three cirrhotic patients with BSIs were enrolled between December 2010 and December 2016 from the Department of Infectious Diseases of the First Affiliated Hospital of Zhejiang University. One hundred twenty-one patients were excluded and 222 cirrhotic patients with BSIs were included (Fig. 1). Demographic and cirrhosis-related clinical data are shown in Table 1. The mean age of patients was 56 ± 13 years; 72.1% of the patients were men. Of the patients, 17.6% had diabetes. The most common etiology of cirrhosis was chronic HBV infection (63.5%), followed by alcohol abuse (22.2%). Of the patients, 61.7% had a history of previous decompensation (PD). Ascites (43.7%) and gastrointestinal bleeding (22.5%) were the most common PD events. Of the patients, 49.5%, 45.9%, and 35.6% received diuretics, antibiotics, and proton pump inhibitor treatment within 3 months before admission, respectively. For the current hospitalization, bacterial infection (44.2%) and ascites (34.2%) were the most common acute decompensation events. The occurrence of organ failure was frequent; liver failure (22.5%) was most common, followed by kidney (10.9%) and heart failure (10.4%).

**Table 1:**
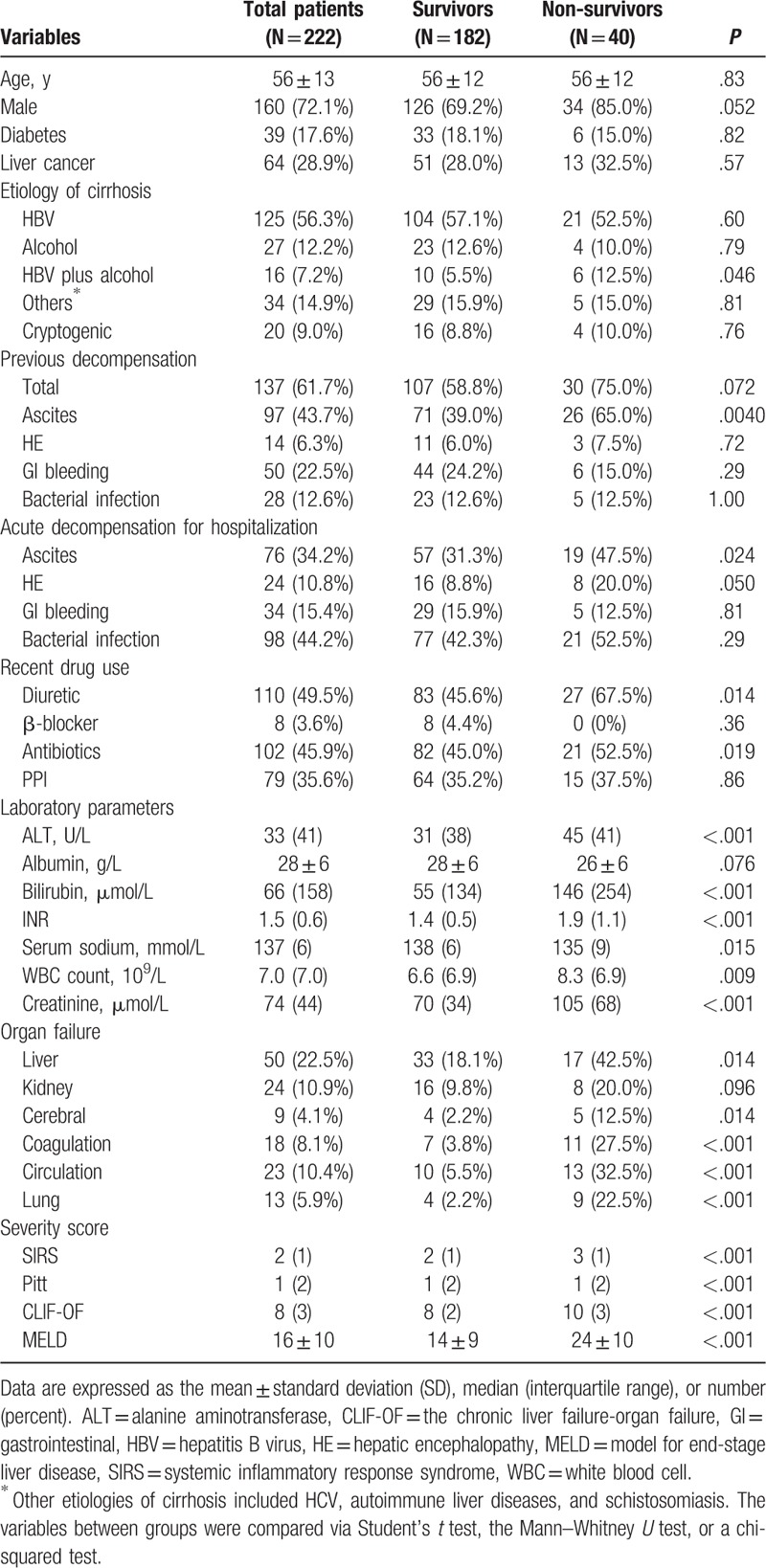
Comparison of demographic and cirrhosis-related data between survivors and non-survivors.

A total of 232 isolates were identified in 222 episodes. BSI-related data are shown in Tables 2 and 3. BSIs occurred a median of 4 days after admission. Regarding the etiology of the BSI, Gram-negative bacteria accounted for 59.9% of cases. Among the Gram-negative bacteria, *Escherichia coli* (21.1%) and *Klebsiella pneumoniae* (20.7%) were most common. Of the isolates, 27.2% were Gram-positive bacteria, of which *Streptococcus* spp. (10.8%) and *Staphylococcus aureus* (7.3%) were most common. Only 12 isolates were caused by fungi, with 9 *Candida* spp. and 3 *Cryptococcus neoformans*. Most episodes (62.5%) were caused by antibiotic-sensitive isolates, with 35.8% multidrug resistant isolates and 1.7% extreme drug resistant. With respect to BSI acquisition, 50.5% of episodes were nosocomial, 31.1% were healthcare-associated, and 18.5% were community-acquired. The most common sources of BSIs were primary (85.6%) and abdominal (10.4%).

**Table 2:**
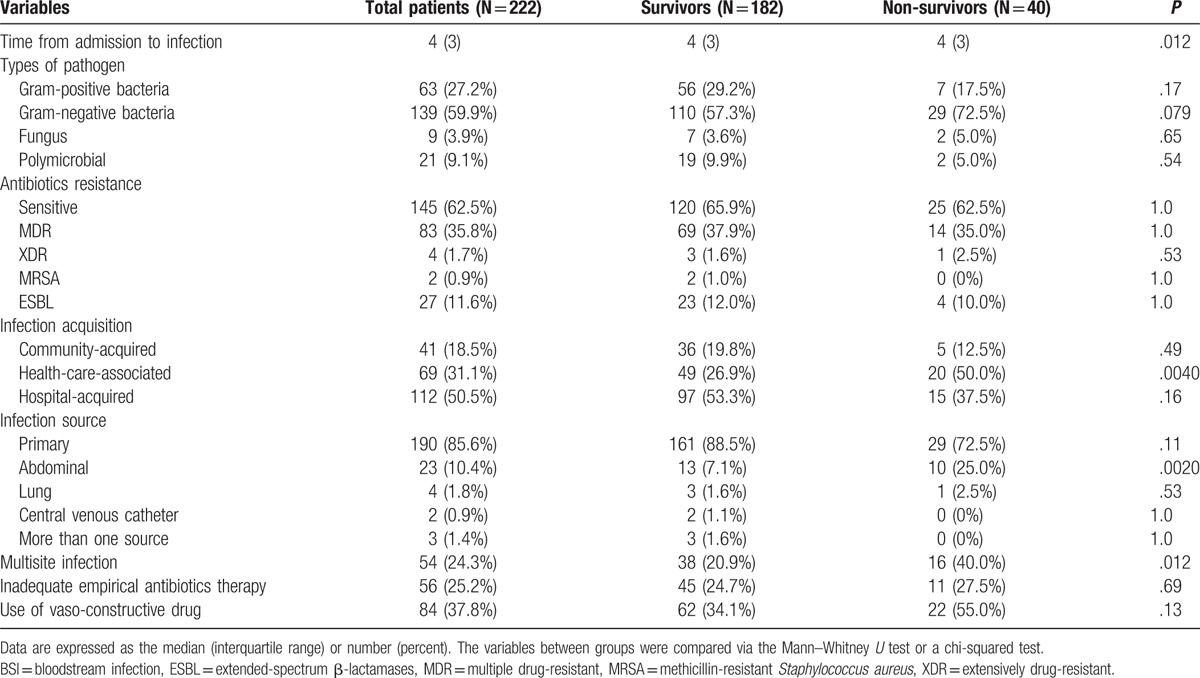
Comparison of BSI-related data between survivors and non-survivors.

**Table 3:**
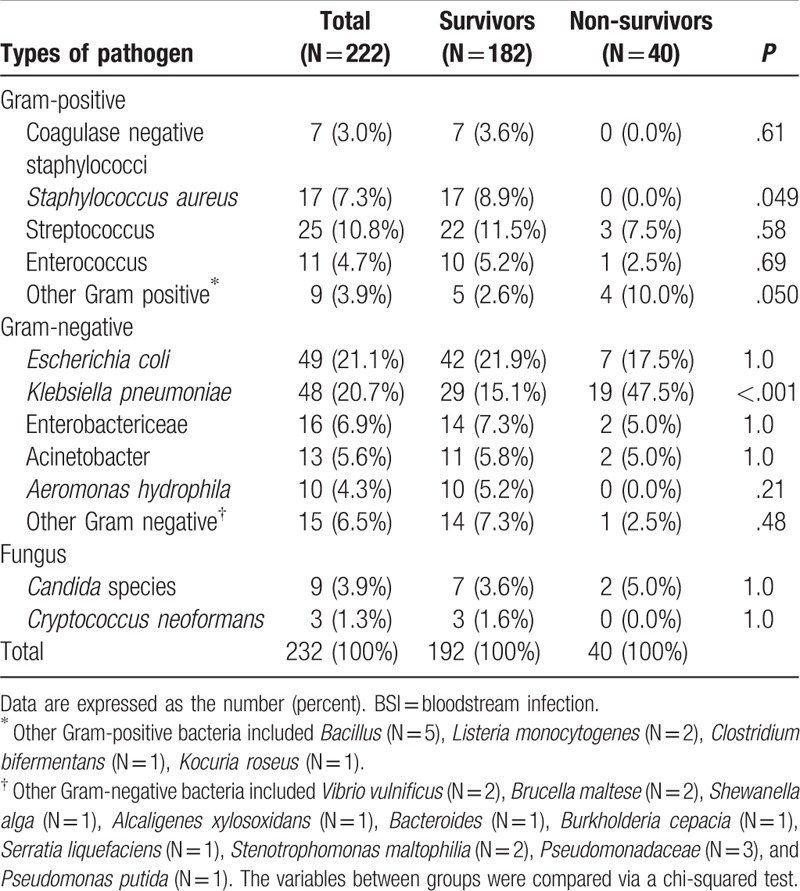
Comparison of etiology of BSI between survivors and non-survivors.

**Figure 2:**
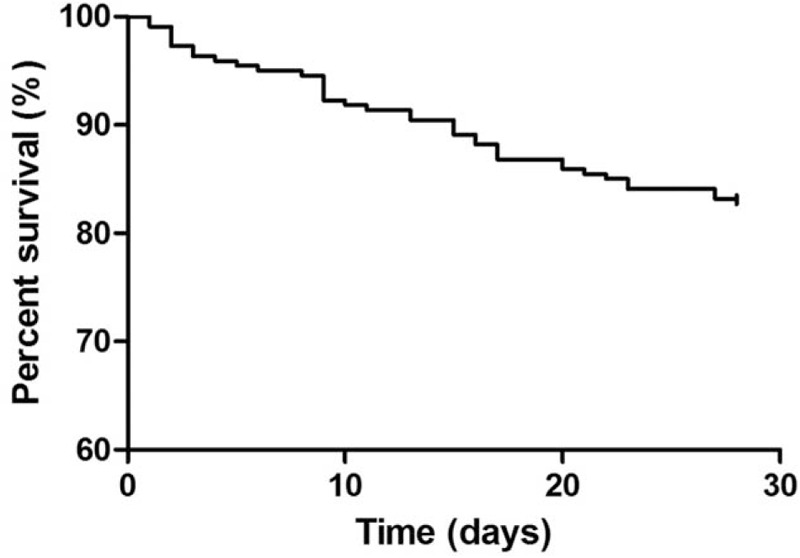
Survival curve of cirrhotic patients with BSI. BSI = bloodstream infections.

Empiric antimicrobial treatment was adequate in 74.8% of episodes. Vasoconstrictive drugs were given in 37.8% of patients. All-cause mortality was 18% on day 28 (Fig. 2).

### 3.2 Predictors of 28-day mortality

Forty deaths were recorded on day 28 after blood was cultured. Male patients were more frequently non-survivors than survivors (85% vs 69.2%, *P* = .052). Non-survivors had more frequent episodes of ascites within recent 3 months (65.0% vs 39.0%, *P* = .004) and had more frequent use of diuretics (67.5% vs 45.6%, *P* = .014) and antibiotics (52.5% vs 45.0%, *P* = .019). Ascites (47.5% vs 31.3%, *P* = .024) and hepatic encephalopathy (20.0% vs 8.8%, *P* = .050) were more frequent acute events for the current hospitalization in non-survivors. Non-survivors generally had a higher incidence of organ failure. With respect to BSI data, non-survivors had more HCA-acquired BSIs (50.0% vs 26.9%, *P* < .001). Multisite infections (40.0% vs 20.9%, *P* = .012) and abdomen-derived BSIs (25.0% vs 7.1%, *P* < .001) were more frequent in non-survivors. With respect to the etiology of BSIs, *K pneumoniae* occurred more frequently in non-survivors (47.5% vs 15.1%, *P* < .001).

An exploratory multivariate analysis of demographics, clinical, and microbiological variables associated with 28-day mortality was performed. By univariate screening analysis, variables introduced in the multivariate Cox hazard model included male sex, history of PD, serum bilirubin, INR, WBC count, presence of hepatic encephalopathy (HE), use of vasoconstrictive drugs, presence of lung failure, multisite infection, and non-primary BSI source. As shown in Table 4, based on multivariate analysis, the variables independently associated with 28-day mortality were male sex (HR = 2.75, 95% CI = 1.10–6.86), INR (HR = 1.76, 95% CI = 1.35–2.30), serum bilirubin (HR = 1.002, 95% CI = 1.000–1.003), circulation failure (HR = 3.56, 95% CI = 1.63–7.79), lung failure (HR = 2.23, 95% CI = 1.09–4.57), and non-primary BSI source (HR = 2.27, 95% CI = 1.09–4.73).

**Table 4:**
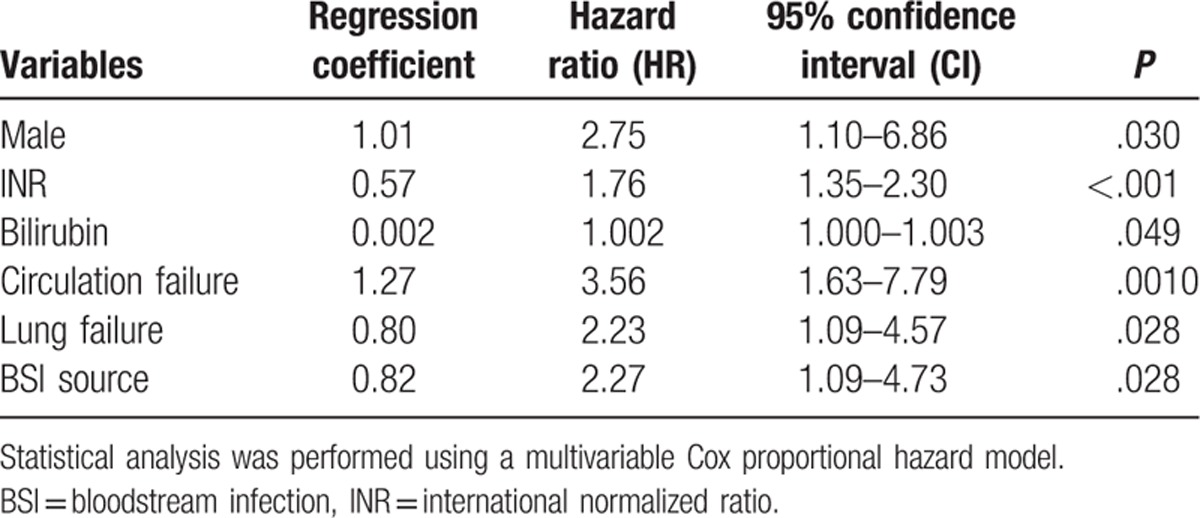
Risk factors associated with 28-day mortality in cirrhosis patients with BSI.

### 3.3 Comparison of scoring systems in predicting 28-day mortality

**Figure 3:**
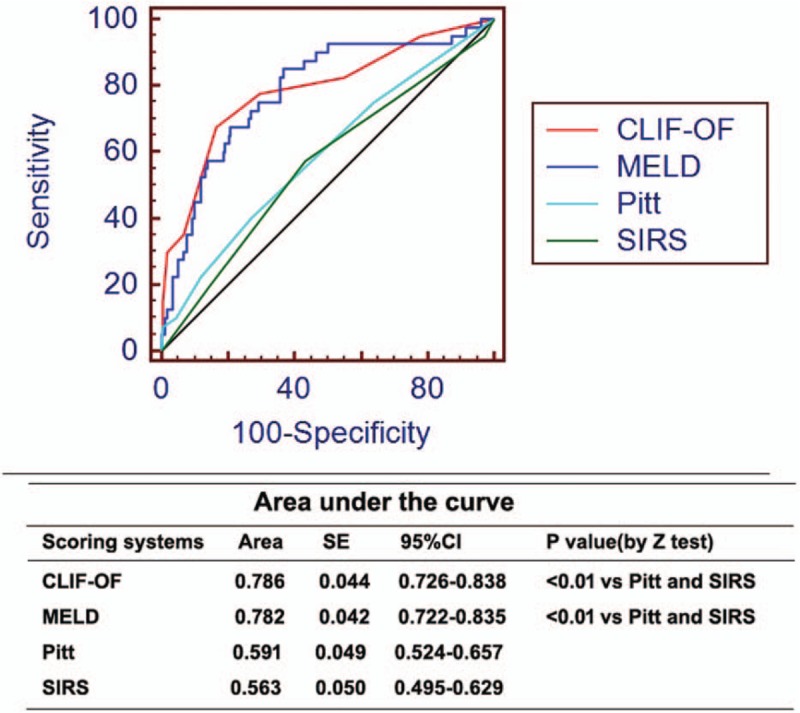
ROC curves of different models in predicting 28-day mortality of cirrhotic patients with BSI. BSI = bloodstream infection, CLIF-OF = the chronic liver failure-organ failure, MELD = model for end-stage liver disease, ROC = receiver operating characteristic curve, SIRS = systemic inflammatory response syndrome.

Four prognostic scoring systems were tested in predicting 28-day mortality of cirrhotic patients with BSIs. The CLIF-OF (0.79) and MELD scores (0.76) had similarly high C-indices, and were significantly higher than the Pitt (0.61) and SIRS scores (0.57). And the CLIF-OF (0.786) and MELD scores (0.782) had significantly higher ROC curves than Pitt (0.591) and SIRS scores (0.637; Table 5 and Fig. 3).

**Table 5:**
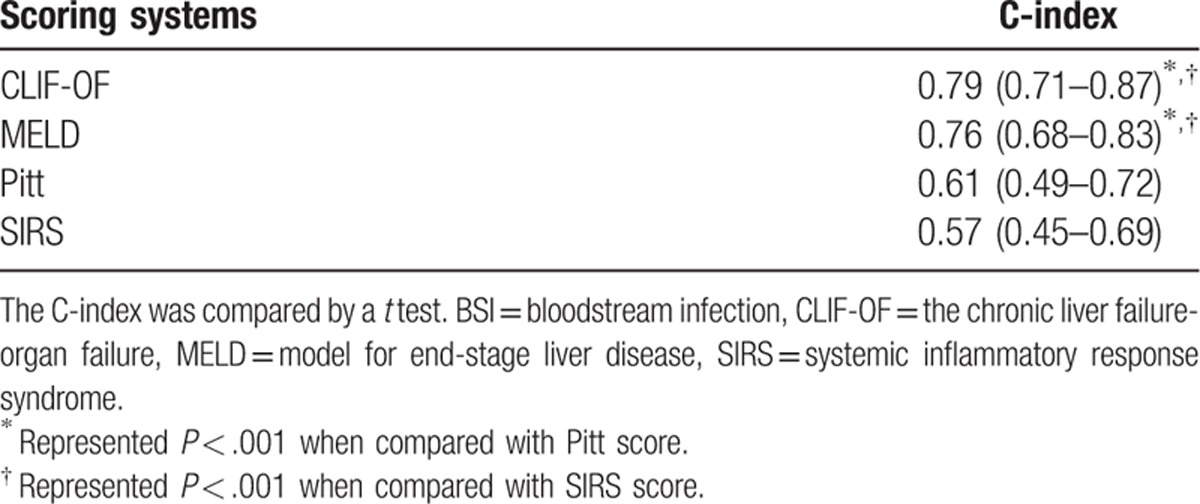
Comparison of C-index in predicting 28-day mortality among different scoring systems in cirrhosis patients with BSI.

## 4 Discussion

The present study investigated the mortality of cirrhotic patients with BSIs, evaluated the risk factors for mortality, and compared the accuracy of different scoring systems for prognostication.

Several studies have reported that inadequate initial antimicrobial therapy increases mortality in patients with BSIs with or without cirrhosis.^[13,17,18]^ In the current study, however, we did not identify an association between inadequate initial antimicrobial therapy and the outcome of cirrhotic patients with BSIs. It should be noted that each patient received antimicrobial therapy within 24 hours after blood was drawn for cultures in our study and the treatment adjustment was conducted according to blood culture results within 3 days. In the current study, inadequate initial treatment occurred in only 25.4% of patients within 24 hours after blood cultures were established. This finding may explain the above discrepancy. The early use of antibiotics may partially explain the relatively low 18% 28-day mortality rate of patients in our study compared with an approximately 30% mortality on day-30 reported by other studies. The early use of antibiotics was supported by a recent study in which early treatment was reported to significantly decrease mortality of patients with SBP.^[19]^ Another reason in support of the early use of antibiotics was that patients in our study had relatively low MELD scores.

With respect to the etiology of BSIs, in agreement with previous studies,^[3,13,18,20,21]^ enterobacteriaceae, especially *Escherichia coli* and *K pneumoniae*, were the major causative pathogens. Altered gut microbiota and increased intestinal permeability may cause an increased risk of enterobacteriaceae BSIs.^[2]^*Streptococcus* spp. were the second common causative pathogens (10.8%) in our study, with a significantly higher incidence than a recent report.^[13]^ This finding might be due to the high frequency of community-acquired/healthcare-associated BSIs in our study. *Candida* spp. were isolated in 3.9% of BSIs. We also found 3 cases of BSIs caused by *C neoformans*.

We evaluated the following 4 scoring systems: CLIF-OF score (organ failure score for cirrhosis); MELD score (liver-specific score); SIRS (inflammation response score); and Pitt score (BSI-specific score). We found that the SIRS and Pitt scores had poor predictive accuracy for prognostication of cirrhotic patients with BSIs. Previous studies have confirmed that cirrhosis is a significant risk factor for mortality in patients with BSIs.^[4,5]^ Therefore, SIRS and Pitt scores, which do not incorporate the parameters measuring the severity of cirrhosis, had significantly impaired predictive accuracy. In addition, cirrhotic patients generally have low WBC counts due to hypersplenism, and often present hypotension and a hyper-dynamic circulatory state in an uninfected state, which further made SIRS criteria inapplicable in patients with cirrhosis.^[2]^ The CLIF-OF score, which was developed to evaluate organ failures in cirrhosis patients, was validated to accurately predict ACLF prognosis in several studies.^[12,22]^ The current study demonstrated that the CLOF-OF score could be used in cirrhotic patients with BSIs as well, in which the incidence of organ failures and ACLF is frequent. The CLIF-OF score had the highest predictive value in the current study, and was slightly better than the MELD score.

The study had some limitations. First, this was a single-center study, in which most patients had HBV-related cirrhosis. Thus, the findings should be further validated in other external cohorts, which were comprised of cirrhosis caused by other etiologies. Second, the dynamic observation of changes in prognostic scores might increase the predictive value; however, longitudinal data were lacking. Third, we did not analyze the long-term prognosis of these patients.

To summarize, cirrhotic patients with BSIs are at high risk for short-term mortality. Our data suggested the CLIF-OF score, as well as the MELD score, unlike the SIRS and Pitt scores, could be used to predict the short-term prognosis of cirrhotic patients with BSIs.
